# Impaired Contextual Fear Extinction Learning is Associated with Aberrant Regulation of CHD-Type Chromatin Remodeling Factors

**DOI:** 10.3389/fnbeh.2015.00313

**Published:** 2015-11-18

**Authors:** Alexandra Wille, Verena Maurer, Paolo Piatti, Nigel Whittle, Dietmar Rieder, Nicolas Singewald, Alexandra Lusser

**Affiliations:** ^1^Division of Molecular Biology, Biocenter, Medical University of InnsbruckInnsbruck, Austria; ^2^Department of Pharmacology and Toxicology, Centre for Molecular Biosciences, Institute of Chemistry and Pharmacy, Leopold-Franzens University of InnsbruckInnsbruck, Austria; ^3^Division of Bioinformatics, Biocenter, Medical University of InnsbruckInnsbruck, Austria

**Keywords:** epigenetics, nucleosome remodeling, amygdala, anxiety behavior, gene regulation

## Abstract

Successful attenuation of fearful memories is a cognitive process requiring initiation of highly coordinated transcription programs. Chromatin-modulating mechanisms such as DNA methylation and histone modifications, including acetylation, are key regulators of these processes. However, knowledge concerning the role of ATP-dependent chromatin remodeling factors (ChRFs) being required for successful fear extinction is lacking. Underscoring the potential importance of these factors that alter histone-DNA contacts within nucleosomes are recent genome-wide association studies linking several ChRFs to various human cognitive and psychiatric disorders. To better understand the role of ChRFs in the brain, and since to date little is known about ChRF expression in the brain, we performed a comprehensive survey of expression levels of 24 ATP-dependent remodelers across different brain areas, and we identified several distinct high molecular weight complexes by chromatographic methods. We next aimed to gain novel insight into the potential regulation of ChRFs in different brain regions in association with normal and impaired fear extinction learning. To this end, we established the 129S1/SvImJ (S1) laboratory mouse strain as a model for compromised contextual fear extinction learning that can be rescued by dietary zinc restriction (ZnR). Using this model along with genetically related but fear extinction-competent 129S6/SvEv (S6) mice as controls, we found that impaired fear extinction in S1 was associated with enhanced ventral hippocampal expression of CHD1 and reduced expression of CHD5 that was normalized following successful rescue of impaired fear extinction. Moreover, a select reduction in CHD3 expression was observed in the ventral hippocampus (vHC) following successful rescue of fear extinction in S1 mice. Taken together, these data provide novel insight into the regulation of specific ChRFs following an impaired cognitive process and its rescue, and they suggest that imbalance of CHD-type remodeler levels, which consequently may lead to changes of transcriptional programs, may be an underlying mechanism involved in impaired fear extinction learning and its therapeutic rescue.

## Introduction

Anxiety and trauma-related disorders are the most prevalent mental disorders in Western societies, with current estimates suggesting that 30% of the population may be afflicted at least once during their lifetime (Kessler et al., 2005; Wittchen et al., 2011). These disorders including phobias, panic, and posttraumatic stress disorder have an important learning component and are often associated with impaired extinction learning, the central mechanism for successful exposure-based therapies (Bouton et al., 2001; Mineka and Zinbarg, 2006). In recent years, it has become increasingly clear that mechanisms that alter the structure and properties of chromatin, sometimes broadly summarized by the term epigenetics, are key players in the regulation of cognitive and emotional processes and thus also of different aspects of fear acquisition, memory, and extinction (reviewed e.g., in Jakovcevski and Akbarian, 2012; Dias et al., 2013; Zovkic et al., 2013; Fischer, 2014; Rudenko and Tsai, 2014; Whittle and Singewald, 2014).

Chromatin remodeling factors (ChRFs) are energy-dependent molecular motor proteins that belong to the SNF2 protein family and can be classified into 23 subgroups according to sequence differences in their ATPase domains and the presence of additional sequence motifs. In mammals, the best studied ChRF subfamilies are the SWI/SNF (switch/sucrose non-fermenting), ISWI (imitation switch), CHD (chromo helicase DNA binding), and the INO80 (inositol auxotroph 80) subfamilies (Lusser and Kadonaga, 2003; Marfella and Imbalzano, 2007; Clapier and Cairns, 2009; Hargreaves and Crabtree, 2011; Piatti et al., 2011). ChRFs use the energy derived from ATP hydrolysis to disrupt and reform histone-DNA contacts. This activity can result in diverse outcomes ranging from the repositioning of nucleosomes along the DNA (sliding), to ejection and assembly of nucleosomes or replacement of canonical with variant histones (Clapier and Cairns, 2009). As a consequence, access to the DNA for transcription factors and the transcription machinery is enhanced or suppressed leading to activation or repression of gene activity.

In contrast to other chromatin-regulatory mechanisms, such as histone modifications or DNA methylation, ChRFs have been given very little attention in brain research. Only recently, several studies have uncovered genetic association of some ChRFs with various intellectual and behavioral disorders (reviewed in Ronan et al., 2013; Krumm et al., 2014; Vogel-Ciernia and Wood, 2014). Given that ChRFs are major regulators of chromatin and transcriptional dynamics and therefore are likely to occupy a central position in the regulation of transcriptional plasticity required for all phases of learning and memory, a better understanding of their role in brain function is highly desirable. Thus, we conducted a broad survey of ChRF expression and investigated their regulation following normal and impaired fear extinction learning. Fear extinction dampens fear expression in response to a conditioned stimulus (CS) or context that no longer predicts aversive events. It is characterized by new learning of a negative relationship between the CS or context and the aversive event while the original fear memory is still in place (reviewed in Johnson and Casey, 2015; Singewald et al., 2015). Investigations into the molecular mechanisms underlying impaired extinction and its therapeutic normalization are important for the development of novel treatment strategies for patients suffering from anxiety and trauma-related conditions since deficient fear extinction can lead to prolonged anxiety and result in stress and anxiety-related disorders. The laboratory mouse strain 129S1/SvImJ (S1) constitutes a convenient model for fear extinction studies as it exhibits compromised fear extinction learning upon cued fear conditioning (Hefner et al., 2008) that can be rescued by dietary zinc (Zn)-restriction (Whittle et al., 2010). Since it was recently shown that the ATP-dependent chromatin remodeling complex nBAF is involved in contextual but not cued fear learning (Vogel-Ciernia et al., 2013), we investigated here potential behavior-associated alterations in ChRF expression levels in contextual fear extinction using S1 mice as well as the genetically related strain 129S6/SvEvTac (S6).

## Materials and methods

### Animals and husbandry

Subjects were male 3-month-old 129S1/SvImJ (S1), 129S6/SvEvTac (S6) mice and C57BL/6 mice (obtained from Charles River and Taconic, Germany) that were housed (4–5 per cage) in a temperature- (22 ± 2°C) and humidity- (50–60%) controlled vivarium under a 12 h light/dark cycle (lights on at 7:00 a.m.). All experimental procedures were approved by the Austrian Animal Experimentation Ethics Board.

### Dietary Zinc restriction (ZnR)

Animals were fed food pellets (ssniff Spezialdiäten) containing low Zn (12.3 mg/kg or 40% of the recommended daily intake requirement; Reeves et al., 1993) or standard food pellets containing normal quantities of Zn (65 mg/kg) as previously described (Whittle et al., 2010). Mice were fear conditioned on standard diet before being placed on ZnR diet for 2 weeks followed by fear expression or extinction training sessions.

### General procedures for contextual fear conditioning

An automated fear-conditioning system (TSE Systems, Bad Homburg, Germany) was used for contextual fear conditioning. Mice were conditioned in a 25 × 25 × 35 cm chamber with transparent walls and a metal rod floor, cleaned with water and illuminated to 300 lux (“context A”). After a 120 s acclimatization period, mice received 2 s scrambled foot shock unconditioned stimulus (US) (0.6 mA) for three times with a 120 s inter-trial interval. After the final US there was a 120 s no-stimulus consolidation period before mice were returned to the home cage. Fear expression or extinction training was performed 14 days later by re-exposing the mice to the conditioning context A for 4 or 16 min, respectively. Freezing was measured as an index of fear (Blanchard and Blanchard, 1969), manually scored based on DVD recordings, defined as no visible movement except that required for respiration, and converted to a percentage [(duration of freezing within the context exposure/total time of the context exposure) × 100] by a trained observer blind to the experimental group.

### Statistical analysis of behavior experiments

The percentage of freezing is presented as mean ± standard error of the mean (SEM). Freezing levels during fear conditioning, expression and extinction training were analyzed using multiple-factor ANOVA with repeated-measures for trial, followed by a Fisher LSD *post-hoc* analysis in case of significant interaction effects. Level of statistical significance was set to *P* < 0.05.

### Brain dissections

Mice were sacrificed 2 h after fear expression or fear extinction training and brains were removed. Amygdala, medial prefrontal cortex (mPFC), dorsal (dHC), and ventral hippocampus (vHC) of both hemispheres were dissected, weighed and snap frozen. Where necessary, dissected regions from two to three animals were pooled. Frozen tissue was stored at −80°C.

### RNA isolation and qRT-PCR

Total RNA was isolated from different brain areas using Tri-reagent (Sigma Aldrich) followed by DNaseI digestion and spin-column clean-up (Zymo Research). Up to 5 μg of RNA were reverse-transcribed using the GoScript Reverse Transcription System (Promega) according to the manufacturer's instructions. Real time PCR was performed in triplicate using POWER SYBR Green PCR mastermix (Applied Biosystems) with 25 ng cDNA and 0.4 μM of target-specific primers. Primer sequences are available upon request. Note that no amplification was obtained for ERCC6, RAD54b, RAD54, and RAD54L2. Data were normalized against *Gapdh*, ΔC_*T*_ values were centered at the median and subjected to hierarchical clustering analysis using Genesis software (Sturn et al., 2002).

### Nuclear extract preparation

Frozen tissues were pulverized using the Cryoprep system (Covaris) and resuspended in five volumes (v/w) homogenization buffer (10 mM Tris-HCl pH 7.9, 5 mM MgCl_2_, 10 mM KCl, 0.34 M sucrose, 1 × protease inhibitor cocktail (Roche), 0.1 mM PMSF, 1 mM DTT). The homogenate was centrifuged for 10 min at 4°C and 2000 × g. The nuclear pellet was carefully resuspended in two volumes (v/w) extraction buffer (15 mM Tris-HCl pH 7.9, 0.25 mM EDTA, 0.43 M NaCl, 10% glycerol, 1 × protease inhibitor cocktail) and incubated on ice for 30 min with gentle mixing. Nuclear extract was obtained by centrifugation at 10,000 × g for 30 min at 4°C.

### Chromatography procedures

Nuclear extract of six brains from 4-week-old male C57BL/6 mice was dialyzed against buffer CB (50 mM Tris-HCl pH 7.9, 100 mM NaCl, 5 mM MgCl_2_, 1 mM EDTA, 10% glycerol, 0.1 mM PMSF, 1 mM DTT) and loaded onto a 1 ml Source15Q anion exchange column (GE Healthcare) on an Äkta Explorer FPLC system (GE Healthcare). After washing with 10 column volumes (CV) buffer CB, proteins were eluted with a 15 CV linear gradient from 100 to 500 mM NaCl in buffer CB. 0.3 ml fractions were collected and subjected to immunoblotting using antibodies against different ChRFs and HDACs. Source15Q fractions containing peak amounts of the analyzed proteins (200–280 mM NaCl) were pooled, applied to a 100 ml Superose 6 size exclusion column (GE Healthcare) and eluted with buffer CB. Two milliliters fractions were collected and proteins were precipitated by addition of 20% (final) trichloroacetic acid (TCA) and incubation for 20 min on ice. Precipitates were collected by centrifugation at 17000 × g for 15 min, washed twice with acetone, dried on ice and dissolved in 1 × SDS loading buffer (75 mM Tris-HCl, pH 6.8, 0.6% SDS, 15% glycerol, and 1.075 M β-mercaptoethanol) for subsequent SDS-gel electrophoresis and western blotting.

### Immunoblotting

Proteins were separated by SDS-PAGE, transferred to nitrocellulose membrane and incubated with antibodies against CHD1 (Proteintech 20576-1-AP; 1:1000), CHD2 (Cell Signaling 4170S; 1:500), CHD3 (Cell Signaling 4241S; 1:500; Novus Biologicals NBP1-51593; 1:1000), CHD4 (Cell Signaling 4245S; 1:500), CHD5 (gift of Michael J. Pazin, HD5A-A Day 77; Potts et al., 2011; 1: 15000), CHD7 (Santa Cruz Biotechnology sc-79207; 1:1000), ATRX (Novus Biologicals NBP1-32851; 1:1000), and Snf2H (Abcam AB3749; 1:500), HDAC1 (Zymed-Invitrogen, 34–8300), HDAC2 (Zymed-Invitrogen, No 34–6400), HDAC3 (Zymed-Invitrogen, 34-7700), and TBP (Millipore 05-1531; 1:250).

### Quantification and statistical analysis

For relative quantification of protein amounts Image Studio Lite software (LI-COR Biosciences) was used. Intensity values were normalized against signals of TBP, which was used as a loading control. For statistical evaluation GraphPad Prism 6.0 software (GraphPad Software, San Diego, CA, USA) was used and Two-way ANOVA with Bonferroni's *post-hoc* test was applied.

### Immunofluorescence microscopy

Twelve weeks old male mice fed with standard food pellets containing normal quantities of Zn (65 mg/kg), were perfused with 4% formaldehyde as described previously (Muigg et al., 2009). Brains were quickly removed and postfixed at 4°C overnight in 4% paraformaldehyde in phosphate buffer and sectioned. Coronal free-floating brain sections of 40 μm thickness were incubated for 30 min in TBS (0.1 M Tris-HCl pH 7.4, 0.9% NaCl) with 1% H_2_O_2_, followed by three washings with TBS. After incubation in 50% formamide/2xSSC (300 mM NaCl, 30 mM sodium citrate tribasic, pH 7) for 2 h at 65°C, sections were washed twice in 2xSSC, treated with 2 M HCl for 30 min at 37°C, washed in 0.1 M borate buffer (pH 8.5) for 10 min followed by three washes in TBS. Samples were blocked for 90 min using 1% bovine serum albumin (BSA) in TBS/0.1% Triton X-100 (TBST/1%BSA). Primary antibody incubations were performed in TBST/1%BSA for 48 h at 4°C with gentle shaking. The following antibodies were used: CHD3 (Novus Biologicals NBP1-51593; 1:1000), Satb2 (Abcam AB92446; 1:800), GABA (Sigma-Aldrich A2052; 1:7000). Following three extensive washing steps with TBST/1%BSA, secondary antibody (anti-mouse ALEXA647, Jackson Immuno Research 715-605-150; 1:500; anit-rabbit CY2, Jackson Immuno Research 711-225-152; 1:500) in TBST/1%BSA was added for 2 h. Sections were again washed 3 times, mounted on microscope slides and dried overnight. ProLong Gold Antifade Mountant with DAPI (Life Technologies P-36931) was applied and the slides were cover-slipped. Microscopy was performed with an Olympus BX51 fluorescence microscope equipped with UPlan Apo 10×/0.40 and PlanApo 60×/1.42 oil immersion objectives. Images were processed using cellSense dimension 1.5 software (Olympus) and Adobe Photoshop CS3.

## Results

### Characterization of ChRF expression patterns and complex formation in the mouse brain

**Figure 1:**
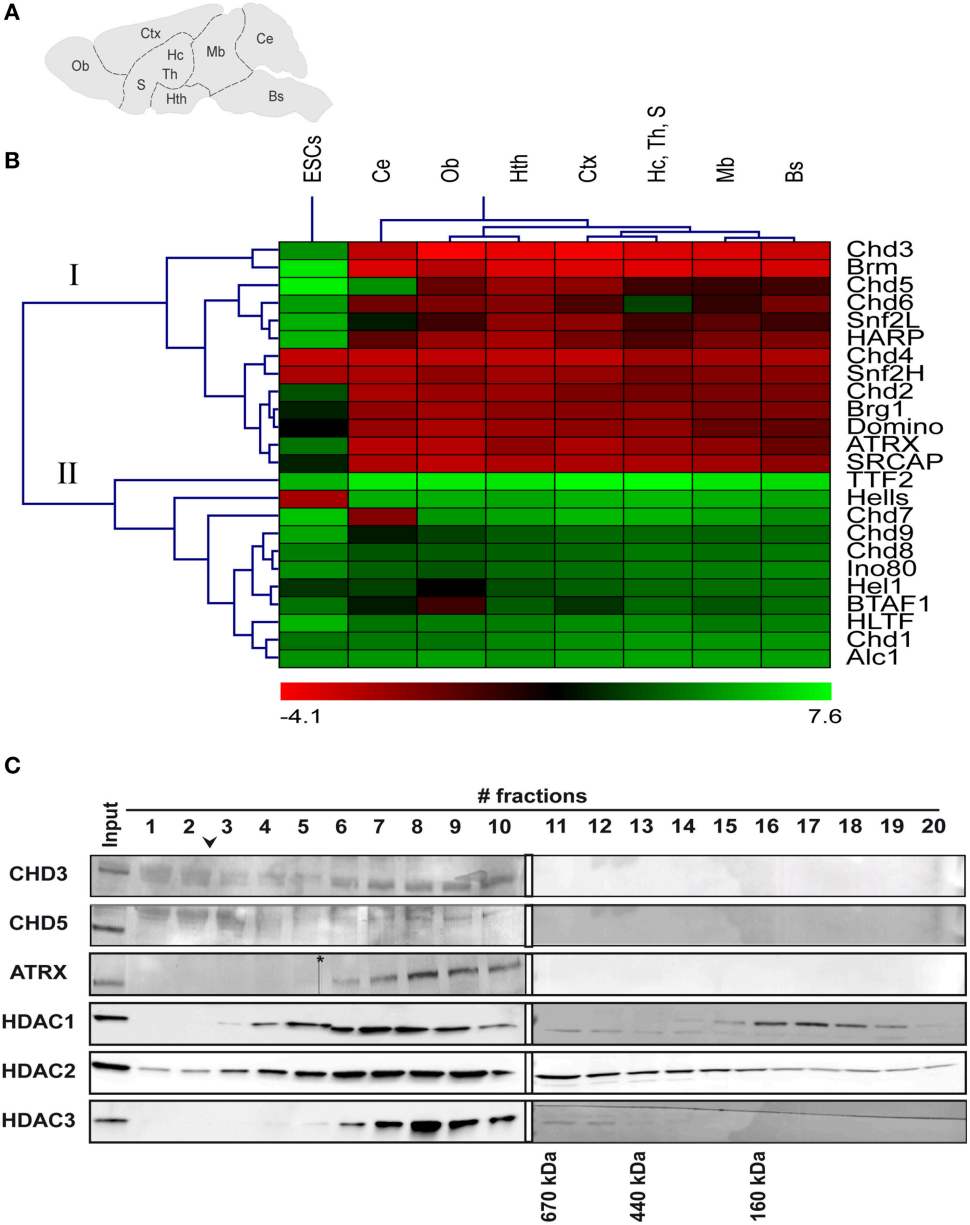
**mRNA expression and protein complex formation of various ChRFs in the brain and in embryonic stem cells (ESCs). (A)** Schematic of regions that were assessed for RNA levels of several ChRFs: Bs, brain stem; Ce, cerebellum; Mb, midbrain; Hth, hypothalamus; Hc, Th, S, hippocampus/thalamus/septum; Ctx, cortex; and Ob, olfactory bulb. **(B)** ChRFs fall into two large expression groups (I + II) in the brain. RT-qPCR results were expressed as ΔC_*T*_ values (reference gene: *Gapdh*) which were then centered at the median and subjected to hierarchical clustering. Red color indicates high expression (negative ΔC_*T*_ value) and green color indicates low expression (positive ΔC_*T*_ value). **(C)** Superose 6 size exclusion chromatography of ChRF peak fractions after anion exchange chromatography followed by immunoblot analysis of CHD3, CHD5, and ATRX as well as HDAC1, 2, and 3. The arrowhead indicates the void volume of the column, the asterisk marks a scanning artifact. Molecular masses of defined marker proteins are indicated at the bottom of their corresponding elution fractions.

To gain an initial overview of the expression of ChRFs in the brain, we performed reverse-transcription qPCR (RT-qPCR) analysis of 24 SNF2-type ATPases belonging to all known mammalian subfamilies (Flaus et al., 2006) from brain stem, cerebellum, midbrain, hypothalamus, hippocampus/thalamus/septum, cortex, and olfactory bulb regions (Figure 1A). Because it has been shown previously that many ChRFs exhibit high expression in mouse embryonic stem cells (ESCs; Efroni et al., 2008), RNA isolated from ESCs was analyzed for comparison. Cluster analysis of median-centered ΔC_*T*_ values revealed two large expression groups (Figure 1B). Group I shows overall higher expression in the brain than in ESCs and comprises 13 ChRFs. CHD3, Brm, and CHD5 of this group displayed the most pronounced enrichment in the brain compared to ESCs. The expression levels of group II factors are generally lower than those of group I in the brain but are similar to the corresponding levels in ESCs. In addition, some factors display specific expression patterns within the brain: for instance, CHD7 is specifically overrepresented in the cerebellum, while CHD5 is depleted from the cerebellum but slightly enriched in the hypothalamus and the cortex; CHD6 is relatively depleted in the hippocampus/thalamus/septum region and BTAF1 is relatively enriched in the olfactory bulb (Figure 1B). With respect to the spatial pattern of ChRF expression the data show that transcript levels deviate most often in the cerebellum from those of other brain regions (Figure 1B).

Next we sought to biochemically analyze ChRF complexes to determine, if different ChRFs form distinct protein complexes in the brain as has been reported for other tissues (Lusser and Kadonaga, 2003; Clapier and Cairns, 2009; Becker and Workman, 2013). In these experiments, we focused on factors of the CHD-subfamily of ChRF (CHD1, CHD3, CHD5) and on ATRX for the following reasons: (i) Antibodies against these factors were commercially available and successfully detected the corresponding proteins in brain protein extracts (Note: we also tested antibodies against CHD2, CHD4, and CHD7 but obtained either no or very faint signals or signals that did not correspond to the calculated size of the protein). (ii) CHD3 and 5 show relatively high expression on the transcript level enabling detection with limited tissue amount. (iii) All these factors have been linked to brain development and/or brain function before (Gaspar-Maia et al., 2009; Bérubé, 2011; Nogami et al., 2011; Potts et al., 2011; Piatti et al., 2015). High salt nuclear extracts were prepared from whole brains; fractionated by Source 15Q anion exchange chromatography and subjected to western blotting. CHD3 and CHD5 segregated clearly from each other and the other two ChRFs in the salt gradient elution, while CHD1 and ATRX coeluted at ~270 mM NaCl (Supplementary Figure 1). To determine if the ChRFs were contained in multisubunit complexes, peak fractions from Source 15Q were pooled and applied to Superose 6 size exclusion chromatography (SEC). CHD3, CHD5, and ATRX eluted with peaks larger than the 660 kDa marker protein thyroglobulin indicating that indeed high-molecular weight complexes are present (Figure 1C). CHD1 signals were too faint after SEC (despite concentration by TCA precipitation) to reliably obtain any size information (data not shown). Since CHD3 and CHD5 are known to form complexes with HDACs in various tissues (Tong et al., 1998; Wade et al., 1998; Xue et al., 1998; Zhang et al., 1998; Potts et al., 2011), we also tested the elution patterns of HDACs1, 2, and 3 on SEC. Signals corresponding to these histone modifying proteins were detected in ChRF-containing fractions. The elution profile of HDAC3 was relatively focused and overlapped well with that of ATRX, CHD5, and CHD3. The profiles of HDAC1 and HDAC2 were broader but still overlapping (Figure 1C). Hence, the elution behavior of CHD3, 5, and ATRX in both anion exchange and SEC suggest that they form distinct high molecular weight complexes in the mouse brain that likely contain HDACs as previously reported for other tissues.

### S6 mice display normal fear extinction learning compared to extinction-deficient S1 mice

Since the building of fear and fear extinction memories requires considerable changes in the transcriptional program of specific brain areas, such as the amygdala, the hippocampus, and the prefrontal cortex (reviewed e.g., in Orsini and Maren, 2012), and it is well known that ChRFs are heavily involved in regulating gene expression at the transcriptional level (Marfella et al., 2006; Hargreaves and Crabtree, 2011; Becker and Workman, 2013), we hypothesized that ChRFs might be critical players in these memory processes and that such a role might be reflected by changes in the amounts of certain ChRFs during different stages of extinction learning. To address this idea, we used two different laboratory mouse strains: The 129S1/SvImJ (hereafter termed S1) mouse strain has previously been shown to exhibit a severe fear extinction deficit when subjected to cued fear conditioning (Hefner et al., 2008). The second strain is the 129S6/SvEvTac (hereafter termed S6) strain, which is closely related to the S1 strain and therefore suitable for comparisons on a molecular level.

**Figure 2:**
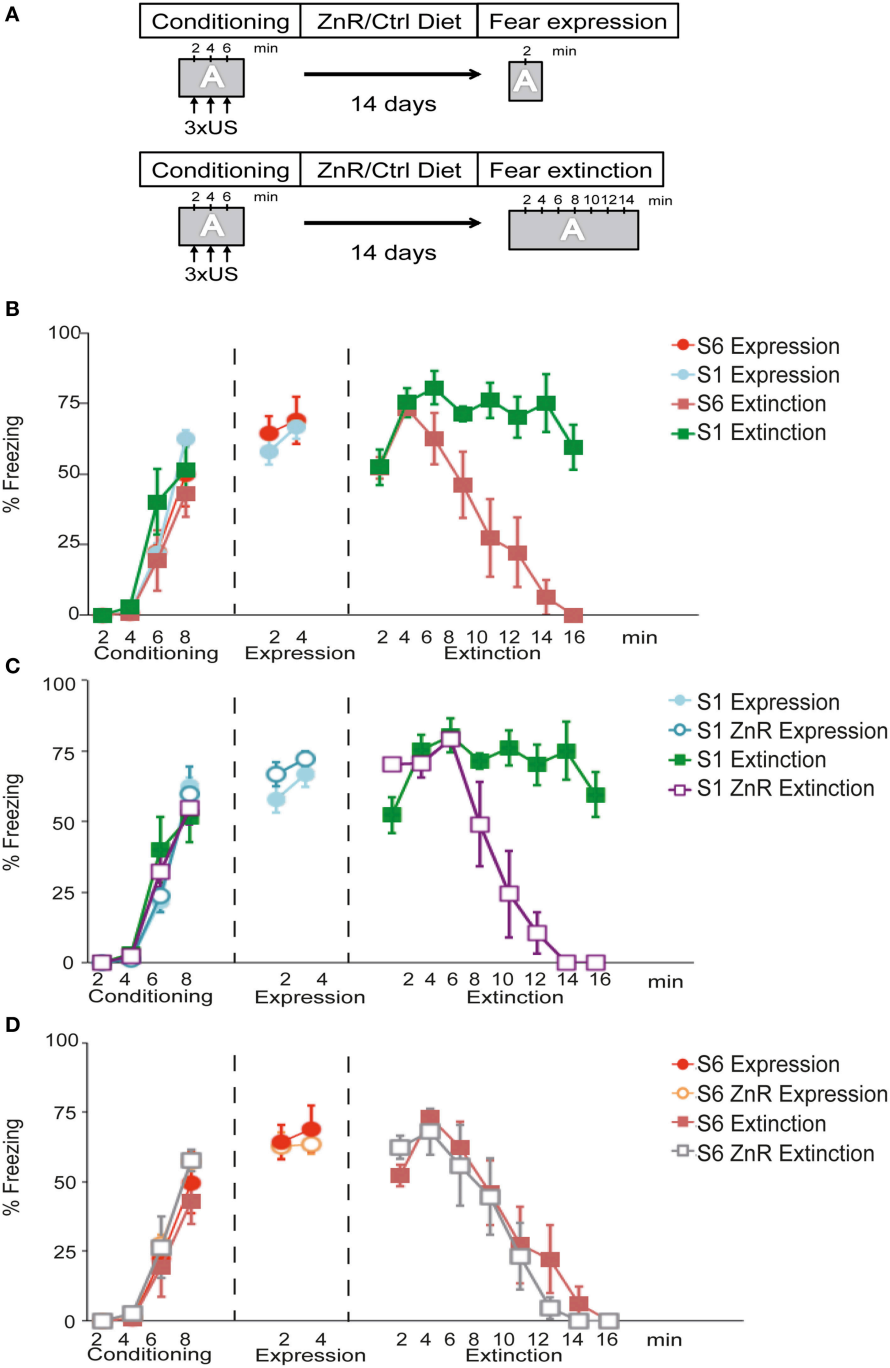
**S1 mice exhibit compromised fear extinction upon contextual fear conditioning that can be rescued by dietary Zn restriction. (A)** Schematic of experimental design. During conditioning, performed in context A (gray box), mice receive 3 mild foot shocks (US) with 120 s non-stimulus intervals. After the final US and a 120 s consolidation period, mice were returned to their home cages. They were either fed a control (Ctrl) or ZnR diet for 14 days before re-exposition to context A and fear expression/extinction monitoring. **(B–D)** Freezing time index during conditioning, fear expression or fear extinction of S1 and S6 mice on Ctrl or ZnR diet. Note, that fear expression and fear extinction, respectively, was tested on separate groups of animals (indicated by different symbols in the graph). Statistical tests were performed comparing all experimental groups and conditions. To improve clarity, however, trend lines were distributed into three separate diagrams. Thus, same-name data groups in **(B–D)** are identical. **(B)** S1 and S6 mice show a significant increase in freezing (*n* = 6/group; *P* < 0.001) during conditioning and similar freezing levels during fear expression training (*n* = 6/group; *P* > 0.05). During fear extinction training, S1 mice displayed significantly higher freezing levels over time than S6 mice (*n* = 5.6 per group; *P* < 0.001). **(C)** S1 mice on ZnR diet show a significant reduction of freezing compared to Ctrl-diet S1 mice (*n* = 5/group; *P* < 0.001), while Zn restriction had no effect on fear expression. **(D)** Zinc restriction does not affect freezing of S6 during conditioning, fear expression and extinction learning (*P* > 0.05; *n* = 6/group).

The first objectives of this experiment were to examine (i) whether S6 mice show normal fear acquisition and extinction behavior and (ii) whether S1 mice fail to attenuate context-dependent fear expression similar to what has been shown before for cued conditioned fear (Hefner et al., 2008). The experimental set-up is depicted in Figure 2A. During conditioning, all experimental groups (*n* = 6/group) showed a significant increase in freezing across conditioning trials [time (freezing to the context) effect: *F*_(3, 60)_ = 100.53, *P* < 0.001], which did not differ between the strains [strain (S6 vs. S1) effect: *F*_(1, 20)_ = 2.51, *P* > 0.05] or groups [group (expression vs. extinction) effect: *F*_(1, 20)_ = 0.00019, *P* > 0.05]. During fear expression, S6 and S1 mice displayed similar levels of freezing to the context [time × strain effect: *F*_(1, 10)_ = 0.41, *P* > 0.05; *n* = 6/group; Figure 2B]. During fear extinction training, there was a significant time × strain interaction for freezing [*F*_(7, 63)_ = 8.29, *P* < 0.001; *n* = 5−6/group; Figure 2B]. *Post-hoc* tests revealed that freezing was significantly lower in S6 than in S1 mice starting after 8 min until the end of the experiment.

It has previously been shown that dietary zinc restriction (ZnR) can successfully induce extinction learning in extinction-impaired S1 mice following cued fear conditioning (Whittle et al., 2010). Therefore, we assessed whether ZnR can also rescue impaired extinction learning in S1 mice in a contextual fear conditioning paradigm (Figure 2C). Freezing levels increased upon three US presentations regardless of group assignment [time (freezing) effect: *F*_(3, 60)_ = 100.78, *P* < 0.001; diet (Ctrl fed vs. ZnR fed) effect: *F*_(1, 20)_ = 0.04, *P* > 0.05; group (expression vs. extinction) effect: *F*_(1, 20)_ = 0.23, *P* > 0.05; *n* = 6/group] (Figure 2C). There was no difference in freezing between Ctrl-fed and ZnR S1 mice during fear expression [time × diet effect: *F*_(1, 10)_ = 0.22, *P* > 0.05; *n* = 6/group], and fear extinction training led to a significant decline in freezing of ZnR S1 mice starting after 8 min until the end of the experiment [time × diet effect: *F*_(7, 56)_ = 15.20, *P* < 0.001; *n* = 5/group] indicating successful fear extinction, while high freezing levels persisted in Ctrl-fed S1 mice (Figure 2C).

### Dietary ZnR does not affect normal fear extinction learning in S6 mice

We also examined the effects of ZnR on fear expression and extinction in extinction-competent S6 mice following contextual fear conditioning (Figure 2D). All groups (*n* = 6/group) showed a similar increase in freezing to the context across US presentations. This was ascertained from ANOVA results that revealed a significant effect on freezing during conditioning of conditioning trials [time (freezing to the context) effect: *F*_(3, 60)_ = 116.72, *P* < 0.001], but not of diet [diet (Ctrl fed vs. ZnR fed) effect: *F*_(1, 20)_ = 1.90, *P* > 0.05] or group [group (expression vs. extinction) effect: *F*_(1, 20)_ = 0.10, *P* > 0.05]. During fear expression, freezing did not differ between Ctrl and ZnR S6 mice [time × diet effect: *F*_(1, 10)_ = 0.49, *P* > 0.05; *n* = 6/group]. Moreover, ZnR did not further promote extinction learning in S6 mice during extinction training, as ANOVA revealed a significant effect of time [*F*_(7, 70)_ = 38.90, *P* < 0.001], but no significant time–diet interaction [*F*_(7, 70)_ = 0.79, *P* > 0.05].

Taken together these results demonstrate that S6 mice display intact contextual fear expression and extinction behavior. By contrast, S1 mice have a deficit in extinguishing context-dependent fear and this deficit can be rescued by dietary Zn restriction.

### Fear extinction involves specific changes in CHD1, CHD3, and CHD5 protein levels

**Figure 3:**
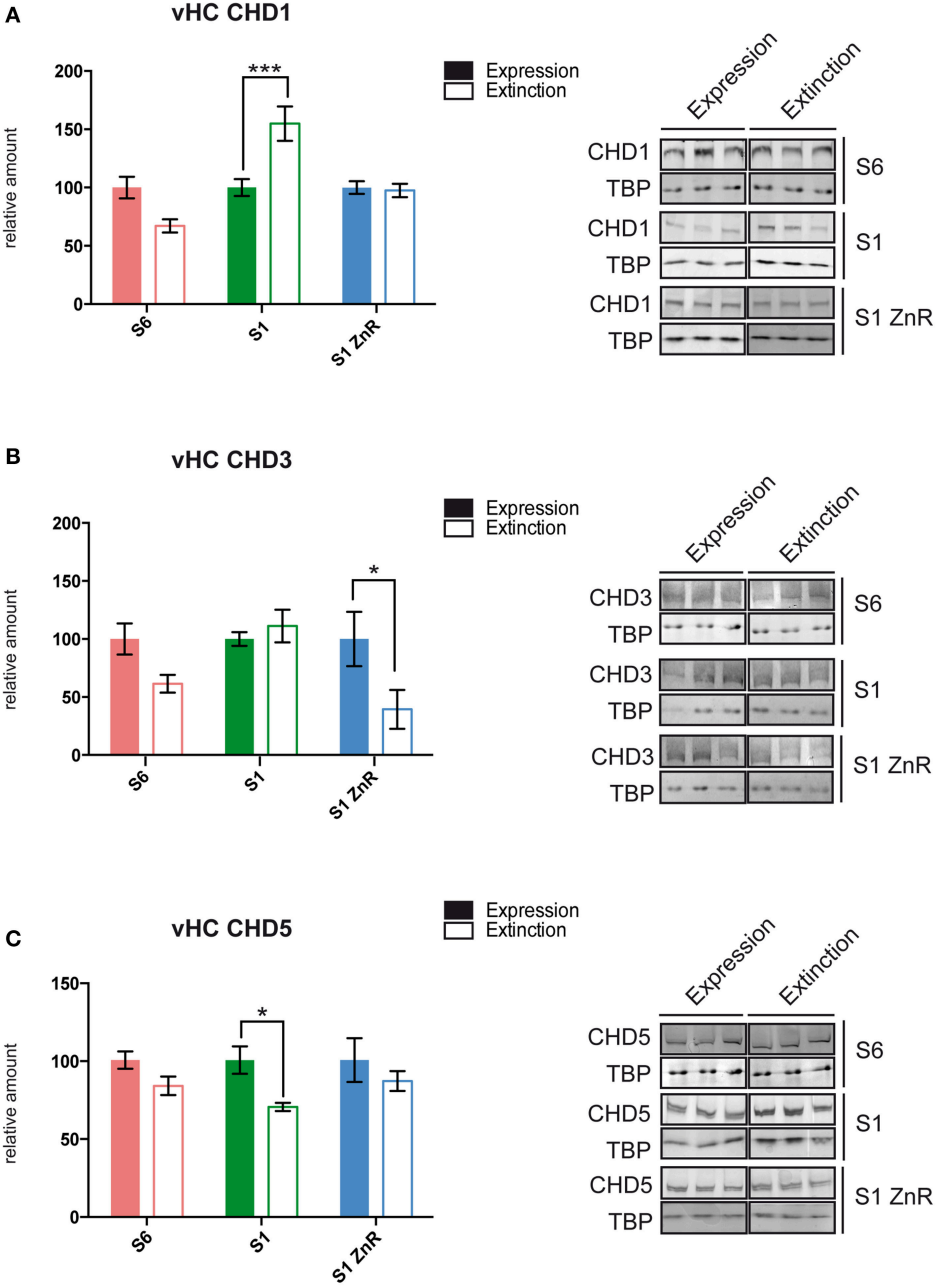
**S1 mice display behavior-dependent aberrant ChRF protein expression in the vHC. (A)** CHD1 showed aberrant up-regulation (S6: *n* = 5; S1 and S1 ZnR: *n* = 6), **(B)** CHD3 failed to become down-regulated (S6: *n* = 5; S1 and S1 ZnR: *n* = 6), and **(C)** CHD5 was down-regulated (S6: *n* = 5; S1: *n* = 4 and S1 ZnR: *n* = 3) following prologed CS exposure in the vHC of extinction-deficient S1 mice. Different brain areas were dissected from S6, S1 and S1 ZnR mice 2 h after contextual fear expression or extinction training, nuclear proteins were extracted and subjected to western blot analysis with antibodies against different ChRFs. Western blot signals were quantified, normalized to TBP and expressed relative to values of the respective fear expression group (left panels). Mean values ± SEM are shown. Statistical significance of protein level differences was determined by Two-way ANOVA with Bonferroni's *post-hoc* test (^*^P < 0.05; ^***^P < 0.005). Right panels, representative western blots of significantly altered proteins are shown.

We used S6, S1, and S1 animals rescued by ZnR diet to dissect brain tissues 2 h after the end of the fear expression or extinction training (Figure 2A) for nuclear protein extract preparation and western blotting with antibodies against the ChRFs CHD1, CHD3, CHD5, CHD7, ATRX, and SNF2h. Specifically, we examined the following brain areas due to their importance for fear learning and memory processes: the amygdala, dorsal hippocampus (dHC), vHC, and the mPFC (Fanselow and Dong, 2010; Marek et al., 2013; Wang et al., 2013; Johnson and Casey, 2015; Singewald et al., 2015; Tovote et al., 2015). While most tested ChRFs did not exhibit significant changes in protein levels between fear expression and fear extinction in the different brain regions and mouse strains (Table 1 and data not shown), we observed significant behavior-dependent regulation of protein levels of CHD1, CHD3, and CHD5 specifically in the vHC. CHD1, which is mostly known as a ChRF regulating active transcription, was upregulated in extinction-impaired S1 mice after unsuccessful extinction training (i.e., prolonged CS exposure). By contrast, when extinction training was successful, such as in S6 and S1 ZnR mice, CHD1 amounts did not vary between short (fear expression) and long (fear extinction) CS exposure (Figure 3A and Table 1). Conversely, the transcriptional co-repressor CHD3 remained unchanged in non-extinguishing S1 mice, but was downregulated after extinction training by 61% in behaviorally rescued S1 ZnR mice. Likewise, a trend toward lower CHD3 amount (−39%, *P* = 0.31) was observed in the vHC of extinction-competent S6 animals following extinction training (Figure 3B, Table 1). Finally, our analyses revealed that CHD5, which is closely related to CHD3 and has predominantly been found as a repressor of transcription, was downregulated by ~30% (*P* = 0.014) in the vHC of S1 mice after non-successful extinction training, while protein levels did not change in extinction-competent S6 and S1 ZnR mice (Figure 3C). Thus, the CHD-family remodelers CHD1, CHD3, and CHD5 show marked deregulation in the extinction-deficient S1 mice that is rescued along with the behavioral defect by dietary restriction of zinc.

**Table 1:** **ChRF expression changes during fear extinction training in different brain areas of extinction-competent S6 and S1 ZnR mice and extinction-deficient S1 mice**.

**Region**	**ChRF**	**S6**		**S1**		**S1-ZnR**	
		**Mean difference (%)**	***P*-value**	**Mean difference (%)**	***P*-value**	**Mean difference (%)**	***P*-value**
vHC	CHD1	−32.95	0.06	54.88	0.0003^***^	−2.49	>0.99
	CHD5	−16.51	0.24	−30.06	0.035^*^	−13.47	0.6737
	CHD3	−38.55	0.31	11.17	>0.99	−60.66	0.021^*^
dHC	CHD1	−27.88	0.56	−40.8	0.12	9.07	>0.99
	CHD5	−37.68	0.38	−34.26	0.38	7.69	>0.99
	SNF2H	−24.00	0.53	−24.95	0.38	−21.32	0.57
Amy	CHD1	9.236	>0.99	−20.59	0.85	39.36	0.14
mPFC	CHD1	23.06	0.51	−22.01	0.82	9.81	>0.99

*vHC, ventral hippocampus; dHC, dorsal hippocampus; Amy, amygdala; mPFC, medial prefrontal cortex. Negative values indicate a decrease*.

* *P < 0.05*,

*** *P < 0.005*.

### CHD3 localizes to excitatory and inhibitory neurons in the hippocampus

**Figure 4:**
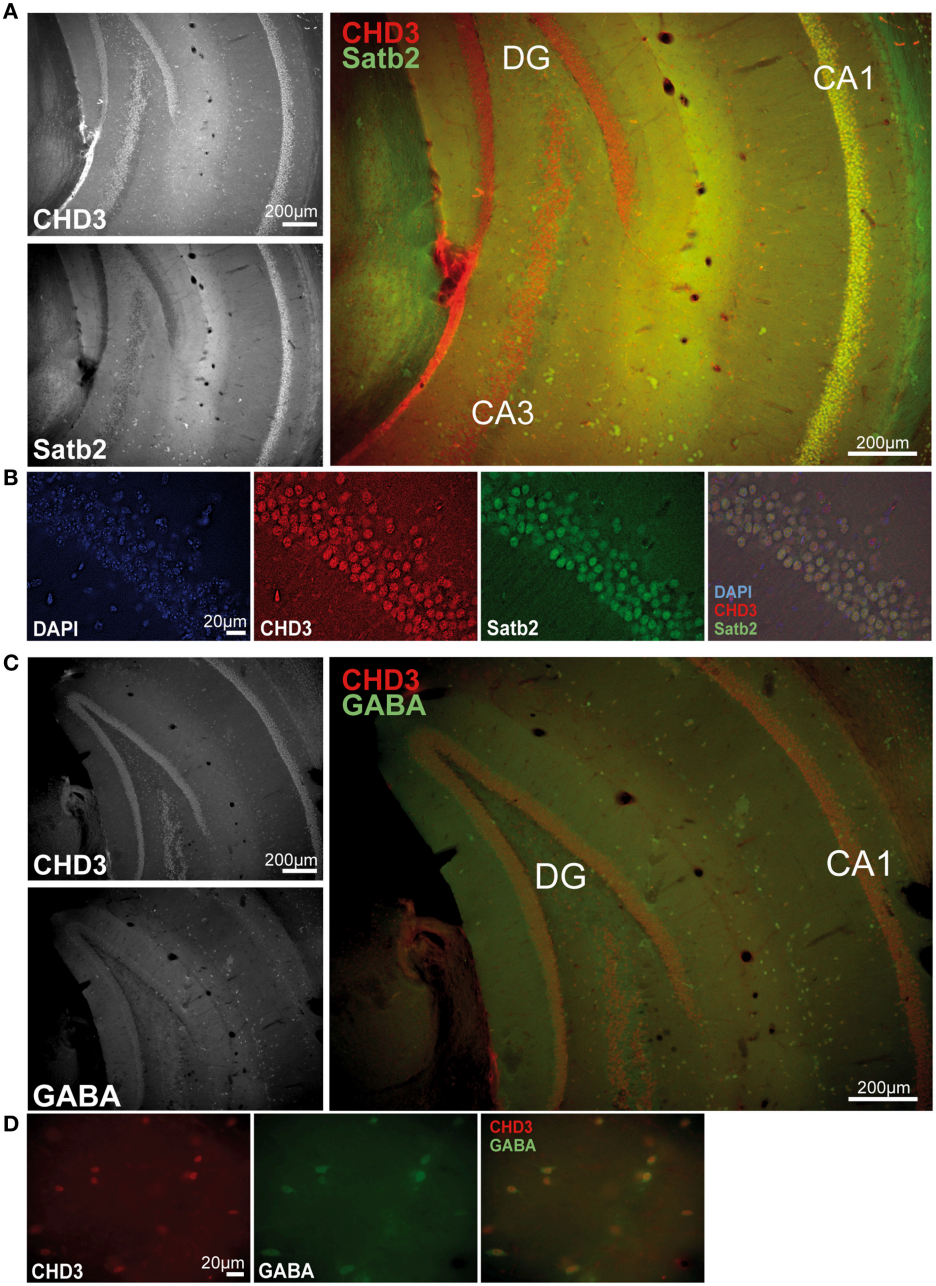
**CHD3 is expressed in all neuronal cell types of the hippocampus**. Mouse brain sections were immunostained with antibodies against CHD3 (red), Satb2 or GABA (green), and DNA was visualized by DAPI staining (blue). **(A)** CHD3-positive cells are found in all layers of the ventral hippocampus. CHD3 is highly expressed in the dentate gyrus (DG), granulae cells and in the pyramidal cell layer of CA1-4. CHD3 colocalizes with Satb2-expressing excitatory neurons in the CA1. **(B)** CHD3 is expressed in nuclei of Satb2-positive excitatory neurons. **(C)** Double staining for GABA and CHD3 in the vHC. **(D)** CHD3 is expressed by inhibitory GABAergic neurons.

Since we have found fear behavior-related differences of CHD1, CHD3, and CHD5 only in the vHC and not in other examined areas, we next asked if these factors show specific subcellular localization in the hippocampus. In a previous study, CHD5 was found to localize to neurons of the hippocampus predominantly in the CA1-CA3 regions but not to glia cells (Bergs et al., 2014). However, CHD1 and CHD3 localization in the hippocampus have not been shown to date. Therefore, we performed immunofluorescence stainings of hippocampal sections with antibodies against CHD3, CHD1, and the excitatory neuron marker Satb2 (Huang et al., 2013) as well as the inhibitory neuron marker GABA. Unfortunately, the CHD1 antibodies were not suitable for tissue stainings regardless of the protocol used. By contrast, robust signals were obtained with antibodies against CHD3 in all nuclei of both the dorsal and vHC including those of Satb2^+^ excitatory and GABA^+^ inhibitory neurons (Figures 4A–D).

## Discussion

ChRFs of the SNF2 family of ATPases are known to be involved in the regulation of diverse mechanisms of DNA metabolism, such as transcription, replication, nuclear architecture, or DNA damage repair (Clapier and Cairns, 2009). While several ChRFs have been studied in the context of brain development, research regarding the role of ChRFs in cognitive and behavioral functions is extremely sparse. The recent discovery of genetic associations between several ChRFs (e.g., BAF complex, CHD8) and intellectual and psychiatric disorders (Ronan et al., 2013; Krumm et al., 2014; Vogel-Ciernia and Wood, 2014), however, underscores the importance of understanding their specific roles in the CNS. Therefore, we performed the first comprehensive analysis of ChRF expression in the brain. The expression levels of 24 members of SNF2-family remodelers revealed distinct patterns for several factors in different brain areas. CHD3 and BRM were the most highly expressed ChRFs in all brain regions, and they exhibited clearly higher levels than in ESCs, which are considered to be particularly enriched for ChRFs (Efroni et al., 2008). BRM is one of the ATPase subunits of nBAF, a neuron-specific chromatin remodeling complex that has recently been implicated in learning and long-term memory formation (Vogel-Ciernia et al., 2013). Its high expression throughout the brain suggests possible functions in general brain physiology. CHD3 is commonly found as part of a multiprotein complex termed NuRD (Nucleosome Remodeling and Deacetylation) which also contains the histone deacetylases HDAC1 and/or HDAC2 (Denslow and Wade, 2007). Although it is currently not known if CHD3 is part of a NuRD-like complex in the brain, we now show that CHD3 is contained in a megadalton complex in the brain that coelutes with HDAC1 and HDAC2 indicative of a NuRD complex. Aside from the widespread expression of CHD3 mRNA in the brain, we detected the protein in Satb2^+^ excitatory and GABA^+^ inhibitory neurons.

High expression levels throughout the brain were also detected for CHD5. This protein is closely related to CHD3 and it was shown to be specifically expressed in mouse brain and testes (Bergs et al., 2014). CHD5 is required for neuronal differentiation during development (Egan et al., 2013) and acts as a tumor suppressor in various cancers (Stanley et al., 2013). Moreover, it was found to form a NuRD-like complex in the brain predominantly containing HDAC2 over HDAC1 (Potts et al., 2011). Our results are consistent with these previous findings, since we also detected CHD5 in a megadalton complex coeluting with HDAC2 in SEC as well as in anion exchange chromatography. We further show that ATRX, another factor that is highly expressed throughout the brain, also forms a high molecular weight complex in brain nuclear extracts. ATRX is recognized as a ChRF that localizes to heterochromatic regions and interacts with a number of transcriptional co-repressors (Ratnakumar and Bernstein, 2013). Mutations in the ATRX gene were found to cause impaired contextual fear memory in mice (Nogami et al., 2011) and α-Thalassaemia/mental Retardation X-linked syndrome in humans (Gibbons et al., 1995). Although in SEC, ATRX perfectly coelutes with HDAC3, the separation profile on anion exchange chromatography argues against a direct association between the two proteins. Taken together, we have characterized the expression levels of most known ChRFs in the brain and we have identified the existence of various high-molecular weight ChRF complexes, including two NuRD-like complexes containing either CHD3 or CHD5.

### Aberrant ChRF protein levels associated with impaired contextual fear extinction

Our studies of ChRFs in the course of fear extinction learning identified three CHD-type remodelers, CHD1, CHD3, and CHD5, to exhibit aberrant protein levels in the extinction-compromised S1 mouse model. Moreover, we found that these changes are restricted to the vHC suggesting a particularly critical role for CHD-type remodelers in this region in the contextual fear extinction process. The role of the vHC in contextual fear conditioning is not entirely clear. Unlike the dHC, the vHC region is directly connected to the amygdala (Pitkänen et al., 2000) and the mPFC (Laroche et al., 2000; Cenquizca and Swanson, 2007; Tovote et al., 2015). Based on evidence from several different studies, it has been suggested that the dorsal hippocampal area is mainly responsible for spatial processing, while the vHC mediates the expression of fear and extinction via projections to the amygdala and the mPFC (Moser and Moser, 1998; Bannerman et al., 2004; Fanselow and Dong, 2010). Importantly, inactivation of the vHC results in impaired extinction of fear (Sierra-Mercado et al., 2011), and it typically interferes with both cued and contextual fear conditioning (Fanselow and Dong, 2010). The S1 mouse model used in this study exhibits fully competent fear learning and expression in response to either a tone stimulus (Hefner et al., 2008) or a context stimulus (Figure 2), but is severely compromised in fear extinction learning for both conditioned stimuli (Hefner et al., 2008; Figure 2). The observed chromatin regulator changes between expression and extinction in the vHC but in none of the other tested areas (dHC, amygdala, mPFC) may suggest that epigenetically balanced regulation of transcriptional programs specifically in the vHC is particularly important for contextual fear extinction learning.

We found that successful but not unsuccessful fear extinction resulted in vHC-specific downregulation of CHD3. These data are consistent with an earlier study in which downregulation of CHD3 mRNA in the HC was found in extinction-competent C57Bl/6 mice using a different contextual fear extinction training protocol (Agis-Balboa et al., 2011). Furthermore, successful fear extinction was found to be associated with decreased levels of HDAC2, which is a partner of CHD3 in the NuRD complex (Wei et al., 2012). NuRD is most often linked to transcriptional repression. However, several studies demonstrating its localization at large numbers of active genes support the possibility that it might have activating as well as repressing roles (Reynolds et al., 2012; Zhang et al., 2012). The targeting of the NuRD complex to specific genes involves interactions with specific transcription factors, such as Ikaros in lymphoid cells or Cdk2ap1 in embryonic stem cells (Kim et al., 1999; Deshpande et al., 2009). It is possible that behaviorally successful fear extinction learning requires regulation of a subset of CHD3-responsive genes in the vHC, which might be compromised in extinction-deficient S1 mice that show no change of CHD3 levels in the course of unsuccessful extinction training.

In contrast to CHD3, we found that unsuccessful extinction training of S1 mice was associated with upregulation of CHD1, which was not observed in extinction-competent S6 and behaviorally rescued S1 mice. CHD1 is generally considered to promote gene activation because it is mostly found at transcriptionally active genes and interacts, for instance, with elongation and splicing factors or the mediator complex (Lin et al., 2011; Park et al., 2014; Siggens et al., 2015). On the other hand, knock-down experiments in ESCs have shown that more genes were upregulated in the absence of CHD1 (including genes involved in neurogenesis) supporting repressive roles for CHD1 (Gaspar-Maia et al., 2009). We have shown previously that a CHD1 N-terminal mutant protein causes defects in ESC differentiation leading to predominant neuronal differentiation (Piatti et al., 2015). Although we show here that the overall expression level of CHD1 in adult mouse brain is rather low, the pronounced deregulation during unsuccessful extinction training in the vHC of S1 suggests that locally and quantitatively restricted expression of this remodeling factor may be important to enable successful extinction learning.

While unsuccessful extinction training in S1 mice led to increased CHD1 levels, the opposite was true for CHD5. Although CHD5 has been shown to form a brain-specific NuRD-like complex, and NuRD complexes are generally regarded as transcriptional repressors, roughly equal numbers of genes were up- and downregulated upon knock-down of CHD5 in primary rat neurons (Potts et al., 2011). Interestingly, this study found that upon CHD5 knock-down, genes classified under the GO term “Behavioral fear response” failed to be upregulated to the same extent over time in culture as observed in control cells. Furthermore, Baf53b and other components of the neuronal nBAF chromatin remodeling complex were upregulated in CHD5 knock-down cells. Baf53b was recently shown to be required for long-term memory of contextual fear (Vogel-Ciernia et al., 2013). In light of these and our data, it is likely that CHD5 is directly and/or indirectly via the nBAF complex involved in the molecular regulation of fear behavior. Thus, it is tempting to speculate that downregulation of CHD5 following impaired fear extinction may result in unbalanced nBAF expression and substantiation of fear memory.

Collectively, our data suggest a possible scenario for the molecular functions of CHD1, CHD3, and CHD5 in compromised fear extinction learning in S1 mice in that a subset of genes that is regulated by CHD3 is not upregulated because CHD3 levels do not decrease following impaired fear extinction. Instead, different subsets of genes that are positively controlled by CHD1 and/or negatively controlled by CHD5 might be activated (e.g., nBAF) thus preventing fear extinction learning and memory. Future gene-expression profiling studies employing the mouse models used in this study combined with brain area specific knock-down of the remodeling factors will be necessary to test these hypotheses. We provide here a first overview of ChRF expression in mouse brain, and we show that CHD-type remodelers are deregulated in a behavior-related manner during contextual fear extinction in the extinction-deficient S1 mouse model.

## Author contributions

AW, VM, PP, NW, NS, and AL conceived the study and designed the experiments; AW, VM, PP, NW, DR performed the experiments; AW, VM, PP, NW, DR, NS, AL analyzed the data and wrote the manuscript.

### Conflict of interest statement

The authors declare that the research was conducted in the absence of any commercial or financial relationships that could be construed as a potential conflict of interest.
