# Supplementary material for: Impaired Contextual Fear Extinction Learning is Associated with Aberrant Regulation of CHD-Type Chromatin Remodeling Factors
Source: Front Behav Neurosci. 2015 Nov 18;9:313. doi: 10.3389/fnbeh.2015.00313 (PMC4649039; doi:10.3389/fnbeh.2015.00313)
Supplement: Supplementary file 1 [file Image1.PDF]

Supplementary Material

Impaired contextual fear extinction learning is associated with aberrant regulation of CHD-type chromatin remodeling factors

Alexandra Wille, Verena Maurer, Paolo Piatti, Nigel Whittle, Dietmar Rieder, Nicolas Singewald* and Alexandra Lusser*

- **Correspondence:** Alexandra Lusser: alexandra.lusser@i-med.ac.at; Nicolas Singewald: nicolas.singewald@uibk.ac.at

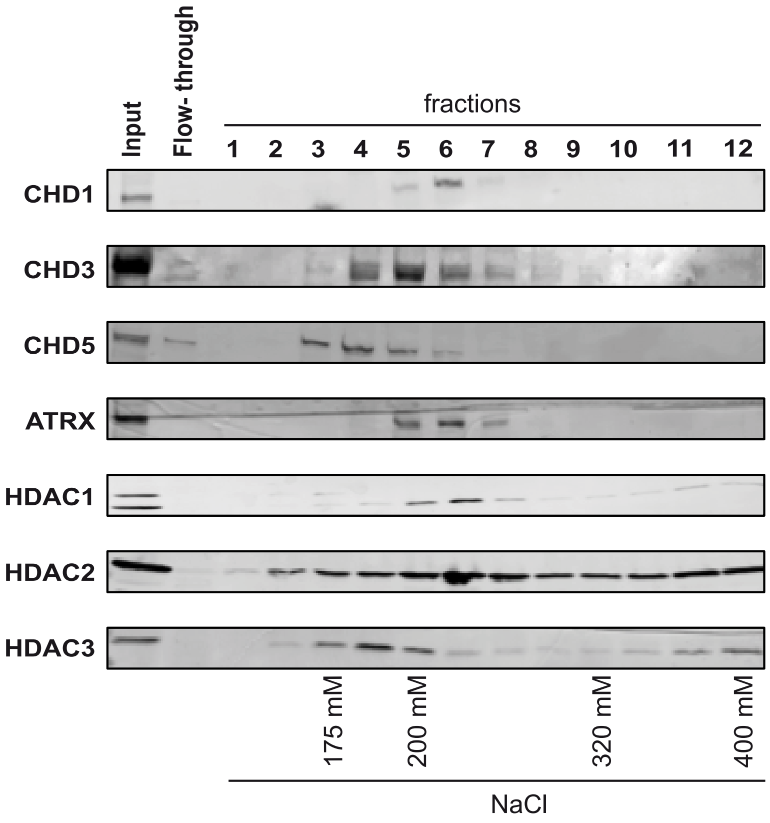

Supplementary Figure 1. Elution profiles of ChRFs and HDACs on anionexchange chromatography. Brain nuclear extract was separated on a Source 15Q column and eluted with a linear salt gradient. 0.3 ml fractions were collected, subjected to western blot and probed with the indicated antibodies.
